# Spatiotemporal evolution and impacts of environment on scrub typhus in northern China, 2006–2019

**DOI:** 10.7189/jogh.15.04202

**Published:** 2025-07-21

**Authors:** Ting Li, Xianjun Wang, Yamei Wang, Chenxin Gu, Liping Yang

**Affiliations:** 1 Department of Epidemiology, School of Public Health, Cheeloo College of Medicine, Shandong University, Jinan, China; 2 Shandong Center for Disease Control and Prevention, Jinan, China

## Abstract

**Background:**

Scrub typhus is a significant public health issue with a global distribution. In northern China, Shandong Province is a major endemic area, but its spatiotemporal patterns and influencing factors remain unclear.

**Methods:**

This study collected data on scrub typhus in Shandong Province from the Infectious Disease Reporting System of the Shandong Center for Disease Control and Prevention between 2006 and 2019. Spatiotemporal evolution analysis combined joinpoint regression, spatiotemporal cluster analysis and standard deviation ellipse. GeoDetector was used to identify the impacts of socioeconomic and natural factors on spatial distribution of scrub typhus. Generalised additive model was applied to explore associations with meteorological variables.

**Results:**

9397 scrub typhus cases were reported in Shandong Province from 2006 to 2019, with an average annual incidence of 0.68 / 100 000, peaking in 2014 (1.53 / 100 000). Cases were concentrated from September to November. Spatiotemporal cluster was mainly in Linyi and Rizhao cities in southern Shandong. The centre of gravity of scrub typhus gradually shifted southeast, and moved back from 2015 to 2019. Nighttime light (*q* = 0.223), normalised difference vegetation index (*q* = 0.197), relief degree of land surface (*q* = 0.230), grassland (*q* = 0.320), and water (*q* = 0.180) were all related with scrub typhus, with *q* indicating the explanatory power of each factor on the spatial distribution of the disease. The strongest relative risks between monthly incidence of scrub typhus and temperature, humidity, precipitation and humidex were 1.528 (lag3), 1.175 (lag3), 1.013 (lag1), and 1.279 (lag3), respectively.

**Conclusions:**

Scrub typhus in Shandong Province was mainly concentrated in Linyi and Rizhao cities. The occurrence of scrub typhus is influenced by various environmental factors. Humidex is a better composite indicator to reflect the impacts of meteorological factors on scrub typhus in northern China. These findings provide scientific evidence to guide prevention and control strategies for scrub typhus. Limitations include potential underreporting in surveillance data and the absence of vector and host information.

Scrub typhus is an infectious zoonotic disease caused by the gram-negative bacterium *Orientia tsutsugamushi* [1]. Symptoms of scrub typhus in humans primarily include fever, rash, and swollen lymph nodes. Severe cases can lead to complications such as multiple organ failure, and even death [2]. Scrub typhus is widely prevalent in the ‘tsutsugamushi triangle’ area, which extends from far eastern Russia in the north, to Pakistan in the west, Australia in the south, and Japan in the east [3]. In recent years, the disease is no longer restricted to this region, showing an expanding geographic distribution and increasing incidence globally [4]. Scrub typhus is estimated to threaten over 1 billion people worldwide yearly, resulting in one million clinical cases [5].

In China, scrub typhus used to be concentrated in the area south of the Yangtze River, but it has gradually spread northwards since the 1980s and appeared in Shandong Province [6]. As scrub typhus continues to spread in northern China, more and more people are at risk of being infected [7]. Shandong Province is a main endemic area in northern China, representing 76.06% of the region's scrub typhus cases between 2010 and 2019 [8]. And due to its widespread occurrence in recent years, it has imposed a heavy burden on public health [9]. Therefore, it is important to focus on Shandong Province to provide a deep understanding of scrub typhus in northern China. Despite the rising burden of scrub typhus in affected regions, current public health policies and surveillance systems in China as well as many other low- and middle-income countries (LMICs), rely on passive case reporting and lack the integration of high-resolution environmental or spatiotemporal data. Such systems are often insufficient for timely risk identification and assessment, spatial targeting of interventions, and resource allocation.

The incidence of scrub typhus may be influenced by various environmental factors. Pan et al. found that natural and socioeconomic factors such as normalized difference vegetation index (NDVI), land use type, and GDP had impacts on the incidence of scrub typhus in the Gannan region [10]. Several studies also demonstrated the positive correlation between meteorological factors like temperature, precipitation, and humidity and the incidence of scrub typhus [11,12]. However, previous studies examining meteorological influences on scrub typhus relied on individual indicators, as seen in regions such as Qingdao [13], Gannan [10], and Anhui [7] in China. In reality, composite indicators such as humidex may better capture the complex effects of meteorological conditions on scrub typhus. Humidex, an index developed by Canadian meteorologists Masterton and Richardson, represents the combined influence of temperature and relative humidity on human comfort and health. It is simpler to calculate [14] and more robust across diverse climate conditions [15] compared to heat index. Whether humidex is a suitable indicator for tropical, mite-borne diseases such as scrub typhus remains uncertain. Therefore, this study aims to explore its utility in this context.

Moreover, existing research on the influencing factors of scrub typhus in Shandong Province has been limited to specific regions. For example, a study conducted in Qingdao City [13] from 2006 to 2018 and our team's previous study on meteorological factors related to scrub typhus in Laiwu District from 2006 to 2012 [16] both focused on localised data. To date, no study has integrated long-term spatiotemporal data across all counties of Shandong Province to assess the effects of socioeconomic, natural, and meteorological drivers including composite indicators.

This study analysed the spatiotemporal evolution of scrub typhus in Shandong Province from 2006 to 2019, as well as the impacts of environment factors on scrub typhus. Additionally, we want to find appropriate composite meteorological indicators, such as humidex to reflect the combined effects of meteorological conditions on scrub typhus well. The results of this study will provide an understanding of how scrub typhus spreads, and identify the related environmental factors influencing the incidence of scrub typhus in northern China. These findings can also support the development of proactive surveillance tools, such as early warning systems and spatial risk maps, which can help guide vector control strategies and more efficiently allocate medical resources in vulnerable regions.

## METHODS

### Study area

The study was conducted in Shandong Province (longitude 114°47.5′-122°42.3′E and latitude 34°22.9′-38°24.01′N), downstream of the Yellow River. Covering an area of 155 800 km^2^, Shandong had a permanent population of 101.2 million by the end of 2023, making it the second most populous province in China. It administers 16 prefecture-level cities and 136 county-level administrative regions. Shandong Province has a warm temperate monsoon climate characterised by concentrated precipitation during hot summers, with short springs and autumns. Its diverse topography, including coastal plains and mountains, creates microclimate conducive to the spread of vector-borne diseases like scrub typhus. Additionally, the province's significant agricultural activities may facilitate disease transmission by providing habitats for disease vectors.

### Data source

Since 2006, scrub typhus was added to the national infectious disease surveillance system. To avoid the impact of the COVID-19 pandemic, data for the period after 2019 were excluded from the present analysis. Therefore, the time period of this study was set as 2006 to 2019. Daily cases of scrub typhus from 1 January 2006 to 31 December 2019 were collected from the Infectious Disease Reporting System maintained by the Shandong Center for Disease Control and Prevention (CDC). Cases have undergone multiple levels of verification and final review by local and provincial CDC. We included only cases from the finalised case report forms. Case types comprised clinically diagnosed cases and laboratory-confirmed cases, with suspected cases excluded from the study. All reported cases were reviewed for completeness and internal consistency. Records with missing or invalid geographic identifiers were excluded. Cases were counted on a county basis, and population data were obtained from the LandScan Global Population Data. Since this study was based on total surveillance data for scrub typhus cases in Shandong Province from 2006 to 2019, a sample size calculation was not required.

Gross domestic product data were derived from the Statistical Yearbook of Shandong Province. Nighttime light data were from the improved time-series DMSP-OLS-like data in China by integrating Defense Meteorological Satellite Program’s Operational Line-scan System (DMSP-OLS) and Suomi National Polar-orbit Partnership’s Visible Infrared Imaging Radiometer Suite (SNPP-VIIRS) [17]. The brightness of nighttime light is an important indicator reflecting human activities and their impacts, and there is a clear relationship between nighttime light and the level of socioeconomic development [18,19]. Normalized Difference Vegetation Index (NDVI) data were obtained from Resource and Environment Science and Data, reflecting the distribution and changes in surface vegetation coverage across different regions. Relief degree of land surface (RDLS) data were obtained from the Geographic remote sensing ecological network platform. It quantifies relative elevation differences and serves as an indicator of landform complexity [20]. Land cover data were provided by the National Cryosphere Desert Data Center and four variables were included in this study: grassland, cropland, water, and impervious. Daily meteorological data were obtained from the National Meteorological Science Data Center, including temperature (°C), relative humidity (%), precipitation (mm) and sunshine (hour). In addition to calculating the monthly total for the precipitation, the remaining variables were calculated for their monthly averages. Additionally, humidex was calculated to assess the combined effects of temperature and relative humidity on scrub typhus incidence. The calculation formula was provided in Equation S1 in the **Online Supplementary Document**.

### Statistical analysis

#### Joinpoint regression

Joinpoint regression is primarily adopted to analyse trend changes in time series. Mathematical statistical analysis methods were used to identify turning points and calculate the annual rate of change (APC), thereby characterising the trend changes in the incidence of scrub typhus across various year intervals.

#### Spatiotemporal evolution analysis

To comprehensively explore the spatial and temporal dynamics of scrub typhus incidence in Shandong Province, we adopted a multi-perspective spatial analytical approach. This study conducted a global spatial autocorrelation analysis, measured by Global Moran's *I*, which ranges from −1 to 1. This index can be used to describe the overall clustering situation across the entire region. Local Moran's *I* for local indication of spatial autocorrelation (LISA) was used to spatially examine the correlation between regions within an area and their neighbouring regions. The cluster maps show four types of aggregations: high-high clusters, low-low clusters, high-low clusters, and low-high clusters. To detect statistically significant space-time clusters and assess their temporal persistence and intensity, we used Kulldorff’s spatiotemporal scanning analysis. The likelihood ratio (LLR) of test statistics was constructed to evaluate whether the number of cases in the window was abnormal. Additionally, the standard deviational ellipse (SDE) method was used to analyse the spatial diffusion direction and the changing trend of the centre of gravity of the scrub typhus incidence from 2006 to 2019. A schematic illustration of the SDE parameters was provided in Figure S1 in the **Online Supplementary Document**. While cluster detection identifies high-risk areas, the SDE captures broader spatial trends such as directional shifts in the geographic centroid of cases. The above analyses were conducted using ArcGIS, version 10.8 (Esri, Redlands, California, USA) and SaTScan version 10.1.2 (Martin Kulldorff, Boston, Massachusetts, USA). Detailed comparative summary of the spatial analysis methods were provided in Table S1 in the **Online Supplementary Document**.

#### GeoDetector model

GeoDetector was used to investigate the driving socioeconomic and natural factors behind the incidence of scrub typhus. Factor detection is used to detect the spatial differentiation of scrub typhus incidence and the explanatory power of different influencing factors on scrub typhus incidence [21]. It is suitable for identifying categorical or stratified spatial influences without assuming linearity or time dependence. The *q* value is used to measure the explanatory power of the independent variables on the incidence of scrub typhus. The *q* value ranges from 0 to 1. The larger the *q* value, the better the independent variables explain the dependent variables. The Geodetector model requires the inputs to be categorical quantities, and the spatial discretisation method used equal interval, geometric interval, natural discontinuity, quantile, standard deviation. The results were compared to find the optimal classification (Table S2 in the **Online Supplementary Document**). GeoDetector model was implemented with *R*, version 4.3.3 (R Foundation for Statistical Computing, Vienna, Austria) using the ‘GD’ packages [22].

#### Generalized Additive Model (GAM)

This study used generalised additive models to investigate the associations between meteorological factors and scrub typhus. We separately incorporated each meteorological variable into GAM and included a variety of covariates to control for constant and time-varying mixed effects. Previous research has found that the impact of meteorological factors on the incidence of scrub typhus can be delayed up to three months [23]. Therefore, three months was utilised to capture the lagged effects in this study. The formulation of GAM is as follows,

*lgE*(*Yi*) *= α + βρi + ns*(*time,df*) *+ ns*(*Zj,df*)

where E(*Yi*) stands for the estimated scrub typhus incidence at time *i*. α is the regression model intercept. β is the exposure response coefficient, which refers to the increase in scrub typhus incidence caused by each increase of 1 unit in influencing factors. *ρi* is the meteorological factor on the *i*-th month. *ns* is a natural spline function (used to control for seasonal and long-term trends). *i* is a date variable. Degree of freedom (*df*) is used to adjust the smoothness of the model. *Zj* is other influencing factors related to the influencing factors studied (used to control the mixing effect of other influencing factors). In our model, by referring to existing research, we selected 6 *df* for the time variable, and 3 *df* for meteorological factors [24,25]. Sensitivity analysis was conducted by adjusting the degree of freedom of long-term trend and seasonality (*df* = 7). Results were expressed as relative risk (RR), both with 95% confidence intervals (CI). The above models were accomplished with R, version 4.3.3 (R Foundation for Statistical Computing, Vienna, Austria) using the ‘mgcv’ packages [26].

## RESULTS

### Epidemiology

**Figure 1:**
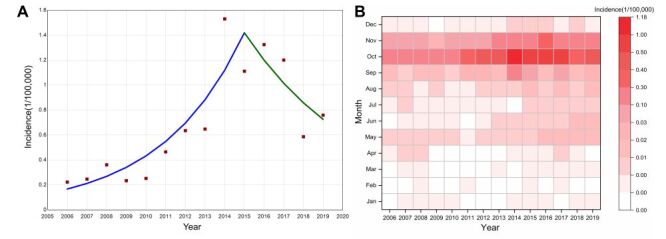
Epidemiological characteristics of scrub typhus incidence in Shandong Province, 2006–2019. **Panel A.** Temporal trends. **Panel B.** Heat map of the monthly composition.

From 2006 to 2019, 9397 scrub typhus cases were reported in Shandong Province. The annual incidence ranged from 0.22 / 100 000 to 1.53 / 100 000, and the average incidence was 0.68 / 100 000. The incidence of scrub typhus was highest in 2014. The joinpoint regression analysis identified one joinpoint in 2015 (**Figure 1**, Panel A). Based on this point, the trend was divided into two segments. During 2006–2015 the incidence trend of scrub typhus exhibited a significantly increasing trend, with an annual percent change (APC) of 26.84% (95% CI = 15.03, 39.86%). The trend from 2015 to 2019 was stable (APC = −15.54%, 95% CI = −33.39, 7.33%). Scrub typhus was concentrated from September to November, accounting for 96.11% of cases, showing a single peak, with sporadic distribution in all other months (**Figure 1**, Panel B).

**Figure 2:**
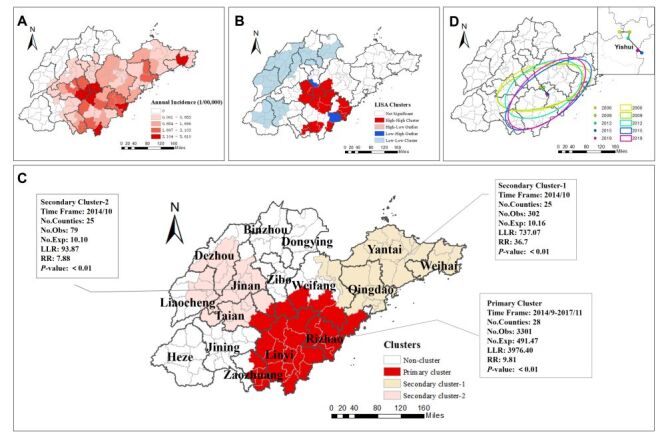
Analysis of scrub typhus incidence in Shandong Province, 2006–2019. **Panel A.** Regional distribution. **Panel B.** LISA cluster maps. **Panel C.** Spatiotemporal scanning clusters. **Panel D.** Standard deviation ellipse and its centre of gravity migration. The ellipse range can encompass 68% scrub typhus cases, and the shift in ellipse centres reflects the temporal migration of the disease’s spatial centre of gravity.

Scrub typhus cases were widespread, cumulatively affecting 13 cities and 87 counties from 2006 to 2019. The disease was concentrated in southern Shandong Province, mainly in the cities of Rizhao, Linyi, and Tai'an. The counties with the highest incidence included Yiyuan (5.61 / 100 000), Mengyin (4.20 / 100 000), and Tancheng (3.88 / 100 000) in Linyi City, Gangcheng (4.26 / 100 000) in Jinan City, and Donggang (3.93 / 100 000) in Rizhao City (**Figure 2**, Panel A). From 2006 to 2019, the number of counties affected by scrub typhus each year were 29, 28, 35, 29, 33, 49, 56, 52, 67, 66, 65, 67,65, and 70, respectively, indicating a continuous expansion in the affected areas (Figure S2 in the **Online Supplementary Document**).

### Spatiotemporal cluster and evolution

Global Moran’s *I* index ranged from 0.17 to 0.54, with *P*-value below 0.01 for each year between 2006 and 2019 (**Table 1**). The incidence of scrub typhus at the county level was clustered, and there was strong spatial dependence across the study area. The LISA analysis confirmed that high-high clusters were concentrated in central Shandong Province (Laiwu, Gangcheng, Xintai, Yiyuan, Mengyin, Linqu, Yishui, and Yinan County), southern Shandong (Linshu, Luozhuang, Lanling, Shizhong, and Yicheng County) and southeastern Shandong (Wulian, Donggang, and Lanshan County) (**Figure 2**, Panel B). There were three clusters (*P* < 0.01) for the period from 2006 to 2019, analysed by spatiotemporal scanning analysis (**Figure 2**, Panel C). Scrub typhus incidence in Shandong Province exhibited notable clustering in both time and space. The primary cluster was centred around Yinan County, mainly in southern Shandong Province. The radius of the cluster was 149.04 km, encompassing 28 counties from 1 September 2014 to 30 November 2017. Relative risk was 9.81, with LLR of 3976.4 (*P* < 0.01). Additionally, two significant secondary clusters were identified (**Figure 2**, Panel C).

**Table 1:** Spatial autocorrelation of scrub typhus incidence in Shandong Province, 2006–2019

Year	Moran's I	Z value	*P*-value
2006	0.17	3.16	<0.01
2007	0.25	4.32	<0.01
2008	0.27	4.45	<0.01
2009	0.25	4.32	<0.01
2010	0.28	4.68	<0.01
2011	0.35	5.95	<0.01
2012	0.39	6.51	<0.01
2013	0.20	3.49	<0.01
2014	0.42	6.80	<0.01
2015	0.36	5.90	<0.01
2016	0.54	8.80	<0.01
2017	0.50	8.22	<0.01
2018	0.46	7.66	<0.01
2019	0.40	6.58	<0.01

The main distribution of scrub typhus was aligned in the southwest-northeast direction (**Figure 2**, Panel D). From 2006 to 2019, the azimuth decreased from 74.359° to 48.971° (Table S3 in the **Online Supplementary Document**). In 2015, the standard deviation ellipse covered the largest area (73 497.874 km^2^). The centre of gravity of the ellipse remained consistently near Yishui County in Linyi City, showing a general trend towards the southeast from 2006 to 2015, and shifting towards the northwest between 2015 and 2019.

### Impacts of environmental factors

Descriptive statistics for environmental variables were provided in supplementary material (Table S4 in the **Online Supplementary Document**). Among the socioeconomic factors, nighttime light was associated with the incidence of scrub typhus (*q* = 0.223), and GDP was not significant (*P* = 0.474). In terms of natural factors, grassland, RDLS, NDVI, and water, were related to scrub typhus, with *q* values of 0.320, 0.230, 0.197, and 0.180, respectively. Among these factors, the *q* value of grassland was the largest, indicating the strongest geographical distribution similarity with scrub typhus incidence and the greatest potential impact. There were no differences between scrub typhus and cropland (*P* = 0.057) or impervious (*P* = 0.660).

**Figure 3:**
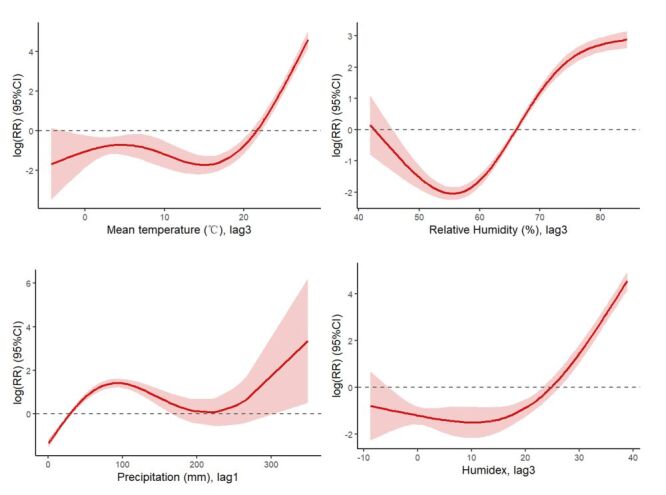
Effects of meteorological factors on scrub typhus incidence. Shaded areas represent 95% confidence intervals.

Apart from sunshine, all other meteorological variables including mean temperature, relative humidity, precipitation, and humidex were associated with the incidence of scrub typhus (*P* < 0.05). The impact of these meteorological factors on the incidence of scrub typhus varied depending on both the variable and the associated time delay (**Table 2**). The forest plot showing RR estimates across lag days was available (Figure S3 in the **Online Supplementary Document**). Mean temperature increased the risk of scrub typhus, with the strongest effect value at lag of three months (RR = 1.528; 95% CI = 1.482, 1.576). Relative humidity was positively correlated with the incidence of scrub typhus at each lag time, and the effect was strongest at lag of three months (RR = 1.175; 95% CI = 1.157, 1.193). The strongest RR of precipitation on scrub typhus was 1.013 (95% CI = 1.011, 1.014) at lag of one month. Humidex was positively correlated with scrub typhus incidence and the effect value was strongest at lag of three months. For each one-unit increase in humidex, the value of scrub typhus incidence increased by 1.279 (95% CI = 1.261, 1.298). The relationship between sunshine and the disease was not significant. In sensitivity analyses, the results remained consistent when the *df* was varied to control for long-term trends (Table S5 in the **Online Supplementary Document**). The influence of each meteorological factor on the incidence of scrub typhus showed nonlinear characteristics (**Figure 3**). Overall, high temperature, humidity, precipitation and humidex could increase the risk of scrub typhus.

**Table 2:** The association of environmental factors with scrub typhus

Factor	q/RR (95% CI)	*P*-value
GDP (10^8^CNY)	0.070	0.474
Nighttime light	0.223	0.009
NDVI (%)	0.197	0.004
RDLS	0.230	0.003
Land cover (km^2^)		
*Grassland*	0.320	0.002
*Cropland*	0.123	0.057
*Water*	0.180	0.009
*Impervious*	0.045	0.660
Mean temperature (°C)		
*Lag 0*	0.990 (0.956, 1.026)	0.589
*Lag 1*	1.092 (1.031, 1.157)	0.003
*Lag 2*	1.265 (1.205, 1.328)	<0.001
*Lag 3*	1.528 (1.482, 1.576)	<0.001
Relative humidity (%)		
*Lag 0*	1.118 (1.103, 1.132)	<0.001
*Lag 1*	1.139 (1.128, 1.151)	<0.001
*Lag 2*	1.143 (1.128, 1.157)	<0.001
*Lag 3*	1.175 (1.157, 1.193)	<0.001
Precipitation (mm)		
*Lag 0*	1.000 (0.995, 1.006)	0.996
*Lag 1*	1.013 (1.011, 1.014)	<0.001
*Lag 2*	1.009 (1.008, 1.010)	<0.001
*Lag 3*	1.009 (1.008, 1.011)	<0.001
Sunshine (h)		
*Lag 0*	0.996 (0.988, 1.004)	0.362
*Lag 1*	0.996 (0.989, 1.003)	0.263
*Lag 2*	1.000 (0.992, 1.008)	0.999
*Lag 3*	0.994 (0.984, 1.005)	0.281
Humidex		
*Lag 0*	0.997 (0.971, 1.023)	0.812
*Lag 1*	1.048 (1.019, 1.078)	0.001
*Lag 2*	1.147 (1.114, 1.181)	<0.001
*Lag 3*	1.279 (1.261, 1.298)	<0.001

CI – confidence interval, NDVI – Normalized difference vegetation index, RDLS – Relief degree of land surface, RR – relative risk

## DISCUSSION

The results of joinpoint regression indicated that the incidence of scrub typhus in Shandong Province showed an overall increasing trend from 2006 to 2015, with a sharp increase between 2013 and 2014. This may be attributed to the improvement in testing capacity or clinical awareness. After 2015, the incidence trend was no longer increasing. Seasonally, scrub typhus cases in Shandong concentrated from September to November. This pattern is consistent with the known seasonal characteristics of scrub typhus in northern China and other temperate regions [7]. It is classified as an autumn-winter type of scrub typhus [27]. Rodents and *L. scutellare* are the main hosts and vectors for the disease in Shandong [28]. *L. scutellare* mites begin to appear in September, peak in October, and decline in November, consistent with the seasonal distribution of scrub typhus cases [29]. Based on the above analysis results, this study will help propose more targeted prevention and control strategies, providing early warning during high-risk periods.

Both spatial autocorrelation and spatiotemporal scanning statistics showed a clustered distribution of scrub typhus in Shandong Province. The clustered area was mainly in the south of Shandong Province, including Linyi, Rizhao and their surrounding areas. The spatial clusters identified in this study closely align with regions classified as high-level habitat quality in a recent land-use and ecological assessment of Shandong Province [30]. These areas are characterised by dense vegetation and well-protected water resources, which provide favourable conditions for the survival and reproduction of chigger mites and their rodent hosts [31,32]. Linyi and surrounding counties are also characterised by intensive agricultural activity [33] and frequent human-vector contact, particularly during the autumn harvest season, which coincides with peak mite activity. Furthermore, SDE analysis showed that the centre of gravity of scrub typhus gradually shifted southeast from 2006 to 2015, and moved back from 2015 to 2019, but it was always mainly distributed around Yishui County in Linyi City. These spatial shifts may also reflect broader environmental and socioeconomic changes, such as land use change, rural development, and public health interventions. After 2015, the implementation of ecological restoration and land-use optimisation policies, such as afforestation and wetland conservation helped alleviate environmental pressure in parts of northern Shandong, leading to signs of partial ecological recovery [34]. These policy-driven changes may have altered habitat suitability and contributed to the observed shift in the disease's spatial distribution. It is necessary to pay attention to the areas of spatiotemporal clusters as well as the direction of migration of the centre of gravity of the disease, so as to carry out precise prevention and control measures for the key regions.

This study revealed that the incidence of scrub typhus is associated with several factors, including the socioeconomic factor of nighttime light and the natural factors of grassland, RDLS, NDVI, and water. Nighttime light, serving as a proxy for socioeconomic level, can influence scrub typhus incidence which is consistent with previous studies [6,13]. In economically developed and densely populated areas, the intensity of nighttime light is stronger, which may imply more outdoor activities and population movement [35]. Moreover, with the development of tourism and urban-rural construction, the habitat of rodents has changed, leading to large-scale migration of rodents and an increased risk of disease incidence [36]. Vegetation and terrain play crucial roles in shaping the ecological niches of both vectors and hosts of scrub typhus. Grasslands and areas with dense vegetation provide favourable microhabitats for chigger mites by maintaining soil humidity and offering shelter from direct sunlight [37]. These environments also support high densities of rodents [38], which serve as primary hosts for chiggers. Terrain variation can also influence local microclimates and vegetation patterns, indirectly affecting the spatial distribution of mite and rodent populations [6]. For instance, areas with moderate elevation tend to retain moisture and support biodiversity, creating ecological conditions conducive to the survival and transmission of scrub typhus.

Climate plays a critical role in the occurrence of infectious diseases, especially vector-borne diseases, through its effects on both vector ecology and human behaviour. Our study confirmed that individual meteorological factors, particularly temperature [7,39,40], relative humidity [41,42], and precipitation [43,44] are associated with scrub typhus incidence, consistent with findings from other endemic regions such as South Korea and India. However, these single-factor associations may not fully capture the interactive effects of climatic variables that jointly drive disease dynamics. To address this, we incorporated humidex, a composite index combining temperature and humidity in our study. Several studies have used humidex to investigate relationships with disease outcomes, such as tuberculosis [45], bacillary dysentery [46], and hand, foot and mouth disease [47]. But to our knowledge, this is the first study using humidex to explore its relevance to scrub typhus. Our findings showed that humidex was significantly associated with scrub typhus incidence, with the strongest effect observed at a three-month lag. Humidex is a more ecologically meaningful measure of environmental suitability, as it incorporates the combined effects of warmth and humidity. And this delayed effect indicates that climatic influences may take time to manifest through changes in the vector-host-environment system. Scrub typhus is transmitted through the bite of infected chigger larvae [37]. Warmer and more humid weather can accelerate development and increase survival, leading to a buildup of infectious larvae after a delay. Rodent reservoir populations may also respond to preceding climatic changes. Human behaviour may further contribute to this delay. In rural Shandong, major agricultural activities such as harvesting typically occur in late summer and early autumn, leading to increased human exposure during high-risk periods. In addition to vector ecology, the disease has an intrinsic incubation period in humans ranging from six to 21 days, which adds further delay between exposure and reported cases [48]. Incorporating transmission dynamic models in future research will help provide a more comprehensive understanding of the disease transmission process.

This study analysed the spatiotemporal evolution and environmental effects on scrub typhus in northern China from 2006 to 2019, identifying high-risk clusters and highlighting the roles of socioeconomic, natural, and meteorological factors. It is essential to implement interventions such as early warning systems for scrub typhus, spatially targeted chigger surveillance especially in high-risk areas, and predictive models designed for policymakers. These evidence-based strategies will improve the sensitivity of surveillance systems and optimise resource allocation for effective scrub typhus prevention and control.

However, this study had several limitations. Scrub typhus case data were obtained from the passive surveillance system, which inevitably led to underreporting. While underreporting may introduce systematic bias, it is likely to have remained relatively consistent over the study period. Therefore, it is reasonable to believe that the impact of the deviation is relatively minor. Additionally, due to the absence of routine entomological surveillance in Shandong Province, data on rodent hosts and chigger vectors were not available for this study. Future research should incorporate targeted field surveillance of vectors and hosts, alongside remote sensing data, to validate and refine the spatiotemporal models in this study.

## CONCLUSIONS

Scrub typhus in northern China was mainly concentrated in Linyi, Rizhao cities and surrounding areas, located in the south of Shandong province. The centre of gravity of scrub typhus was gradually shifting southeast from 2006 to 2015, and moving back from 2015 to 2019. The occurrence of scrub typhus could be influenced by various environmental factors, such as nighttime light, grassland, RDLS, NDVI, and water. Moreover, this study identified the effect of composite indicator, humidex, on the incidence of scrub typhus, optimising previous studies that focused only on individual meteorological variables.

## Additional material


Online Supplementary Document
