# Supplementary material for: Spatiotemporal evolution and impacts of environment on scrub typhus in northern China, 2006–2019
Source: J Glob Health. 2025 Jul 21;15:04202. doi: 10.7189/jogh.15.04202 (PMC12278688; doi:10.7189/jogh.15.04202)
Supplement: Online Supplementary Document [file jogh-15-04202-s001.pdf]

Supplement to: Li T, Wang X, Wang Y, Gu C, Yang L. Spatiotemporal evolution and impacts of environment on scrub typhus in northern China, 2006–2019. J Glob Health. 2025;15:04202.

Equation S1. The formula of humidex.
Table S1. Summary of spatiotemporal methods applied to identify spatiotemporal evolution of scrub typhus in Shandong Province.
Table S2. The selection of specific discretization method and interrupt count for data in Geodetector.
Table S3. Ellipse parameters of standard deviation of scrub typhus incidence in Shandong Province from 2006 to 2019.
Table S4. Descriptive analysis of influencing factors of scrub typhus.
Table S5. Results of sensitive analysis by changing the degree of freedom of long-term trend and seasonality (*df*=7).
Figure S1. Schematic diagram of standard deviational ellipse (SDE) and its key parameters.
Figure S2. Regional distribution of scrub typhus incidence in Shandong Province, 2006-2019.
Figure S3. Forest plot of relative risks (RR) and 95% confidence intervals for meteorological variables at different lags.

Equation S1. The formula of humidex:

$$Humidex = Tem + \frac{5}{9}\{6.112 \times 10^{\left(\frac{7.5\times Tem}{237.7+Tem}\right)} \times \frac{Hum}{100} - 10\},$$

where *Tem* is temperature; *Hum* is relative humidity

Table S1. Summary of spatiotemporal methods applied to identify spatiotemporal evolution of scrub typhus in Shandong Province.

Method	Dimension	Purpose
Global spatial autocorrelation(Global Moran's I)	Space (global)	To evaluate whether global spatial autocorrelation exists
Local indication of spatial autocorrelation (LISA)	Space (local)	To identify localized clusters and classify cluster types
Kulldorff's spatiotemporal scanning	Space + time	To detect high-risk clusters over time and space
Standard deviation ellipse(SDE)	Space + time (directional)	To examine the directional trend and movement of case distribution

Table S2. The selection of specific discretization method and interrupt count for data in Geodetector.

	Variable	Discretization method	Number of intervals
X1	NDVI	quantile	10
X2	RDLS	natural	10
X3	Area of grassland (km^2)	geometric	8
X4	Area of cropland (km^2)	quantile	9
X5	Area of water (km^2)	quantile	10
X6	Area of impervious (km^2)	quantile	10
X7	GDP	quantile	10
X8	Nighttime light	geometric	10

Table S3. Ellipse parameters of standard deviation of scrub typhus incidence in Shandong Province from 2006 to 2019.

Year	CenterX	CenterY	XStdDist/km	YStdDist/km	Rotation	Shape area/km^2	XStdDist/ YStdDist
2006	118°27'	36°2'	201.174	99.746	74.359	63033.869	2.017
2009	118°36'	36°1'	218.730	89.198	74.339	61285.346	2.453
2012	118°34'	35°57'	187.949	109.699	61.681	64767.827	1.713
2015	118°46'	35°47'	230.084	101.692	53.129	73497.874	2.263
2019	118°42'	35°49'	212.423	106.238	48.971	70890.925	1.999

Table S4. Descriptive analysis of influencing factors of scrub typhus.

	Variable	Mean	SD	Min	Percentiles			Max
					25%	Median	75%	
X1	NDVI	0.700	0.087	0.385	0.668	0.723	0.762	0.802
X2	RDLS	0.094	0.111	0.002	0.032	0.054	0.107	0.649
X3	Grassland (km^2)	22.671	44.688	0.001	0.111	2.168	23.163	288.464
X4	Cropland (km^2)	823.769	436.294	1.588	551.753	836.003	1108.568	2535.187
X5	Water (km^2)	34.305	88.649	0.082	4.968	14.682	30.079	916.208
X6	Impervious (km^2)	207.107	87.409	26.618	147.493	201.784	253.442	495.112
X7	GDP (10^8CNY)	6.838	3.900	2.300	3.800	5.650	8.400	20.900
X8	Nighttime light	14.837	12.633	3.159	7.273	9.587	17.634	62.335

X9	Mean temperature (°C)	13.883	9.906	-4.314	4.951	14.997	22.758	28.100
X10	Relative Humidity (%)	64.88	9.790	41.85	57.94	63.25	72.32	84.40
X11	Precipitation (mm)	56.76	67.284	0.52	10.31	28.61	75.99	349.23
X12	Sunshine (h)	42.51	42.510	65.54	154.78	192.32	218.68	300.70
X13	Humidex	14.385	14.161	-9.780	2.313	15.624	28.388	38.902

Table S5. Results of sensitive analysis by changing the degree of freedom of long-term trend and seasonality (*df*=7).

Variables	Effect	*P* value
Mean temperature (°C)		
Lag 0	0.991 (0.956, 1.026)	0.605
Lag 1	1.093 (1.032, 1.159)	0.003
Lag 2	1.270 (1.208, 1.336)	0.002
Lag 3	1.545 (1.497, 1.594)	<0.001
Relative Humidity (%)		
Lag 0	1.121 (1.106, 1.136)	<0.001
Lag 1	1.138 (1.127, 1.150)	<0.001
Lag 2	1.144 (1.129, 1.159)	<0.001
Lag 3	1.168 (1.150, 1.186)	<0.001
Precipitation (mm)		
Lag 0	1.000 (0.995, 1.005)	0.999
Lag 1	1.012 (1.011, 1.014)	<0.001
Lag 2	1.009 (1.008, 1.010)	<0.001
Lag 3	1.009 (1.008, 1.011)	<0.001
Sunshine (h)		
Lag 0	0.996 (0.988, 1.004)	0.365
Lag 1	0.996 (0.989, 1.003)	0.256
Lag 2	1.000 (0.992, 1.008)	0.988
Lag 3	0.995 (0.985, 1.005)	0.293
Humidex		
Lag 0	0.997 (0.971, 1.024)	0.812
Lag 1	1.048 (1.019, 1.078)	0.001
Lag 2	1.148 (1.114, 1.182)	<0.001
Lag 3	1.283 (1.265, 1.302)	<0.001

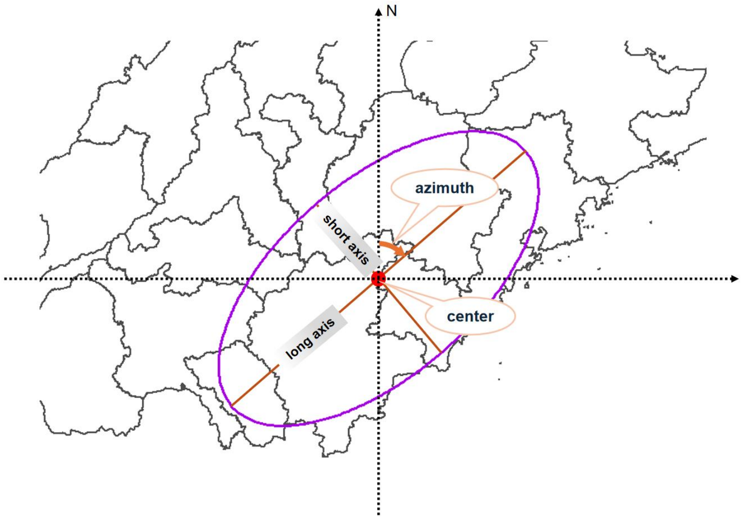

Figure S1. Schematic diagram of standard deviational ellipse (SDE) and its key parameters.

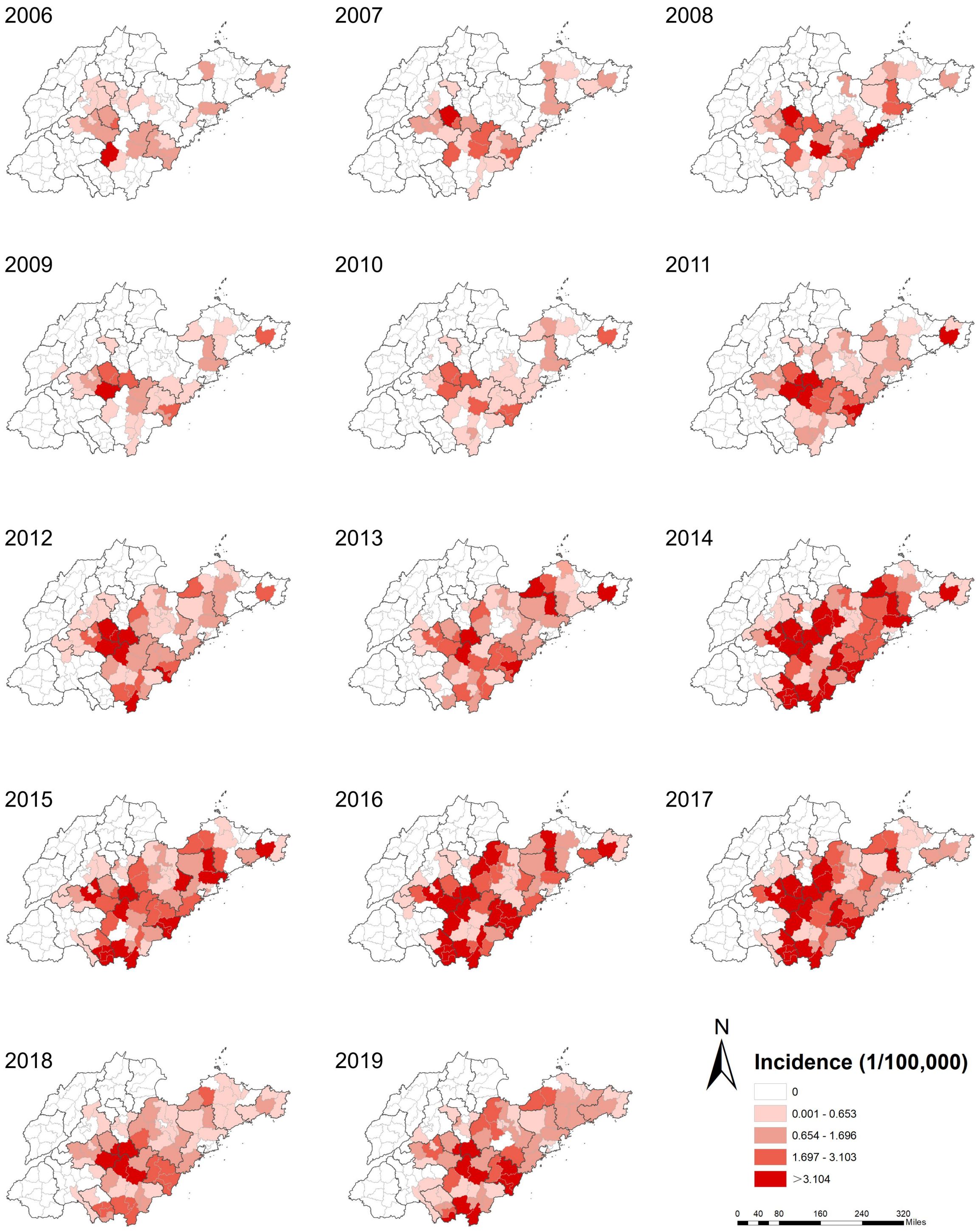

Figure S2. Regional distribution of scrub typhus incidence in Shandong Province, 2006-2019.

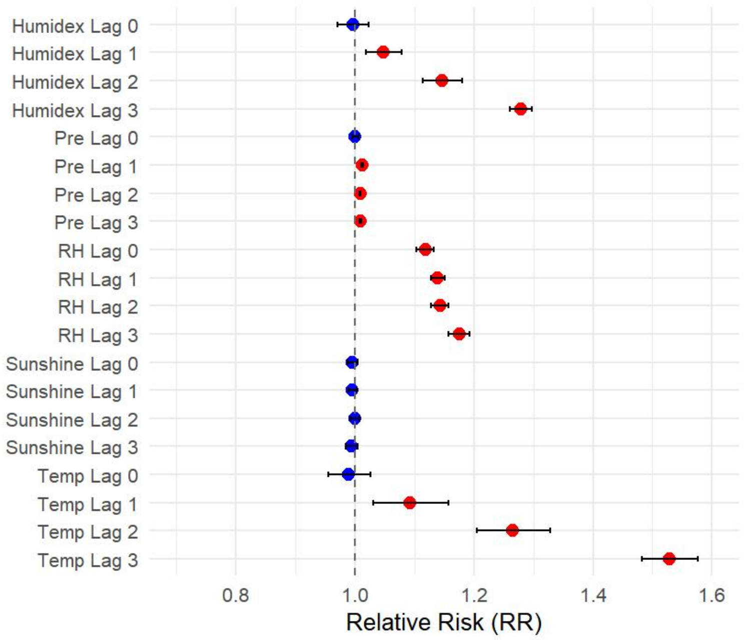

Figure S3. Forest plot of relative risks (RR) and 95% confidence intervals for meteorological variables at different lags.
